# Low Level of First Morning Urine Cardiac Troponin I: A Specific Hallmark of Aortic Stenosis Severity

**DOI:** 10.3390/jcm13092472

**Published:** 2024-04-24

**Authors:** Tomo Svaguša, Marko Žarak, Dubravka Šušnjar, Savica Gjorgjievska, Josip Varvodić, Nikola Slišković, Gloria Šestan, Marko Kušurin, Ingrid Prkačin, Igor Rudež

**Affiliations:** 1 Department of Cardiovascular Disease, Dubrava University Hospital, 10000 Zagreb, Croatia; svagusa.tomo@gmail.com; 2 Clinical Department of Laboratory Diagnostics, Dubrava University Hospital, 10000 Zagreb, Croatia; 3 Faculty of Pharmacy and Biochemistry, University of Zagreb, 10000 Zagreb, Croatia; 4 Department of Cardiac and Transplant Surgery, Dubrava University Hospital, 10000 Zagreb, Croatia; dubravka.susnjar@gmail.com (D.Š.); gjsavica@gmail.com (S.G.); josip.varvodic@gmail.com (J.V.); sliskovic.nikola1@gmail.com (N.S.); gloria.sestan@gmail.com (G.Š.); kusurin.marko@gmail.com (M.K.); rudi@kbd.hr (I.R.); 5 Department of Internal Medicine, Merkur University Hospital, 10000 Zagreb, Croatia; 6 School of Medicine, University of Zagreb, 10000 Zagreb, Croatia

**Keywords:** cardiac troponin I, cardiac troponin T, aortic stenosis, urine

## Abstract

**Background**: It has recently been shown that cardiac-specific troponin I concentrations in first morning urine samples can be measured with commercially available tests. Due to their accumulation in the first morning urine, scientific papers indicate a potential predictive value for cardiovascular diseases. Therefore, the aim of this study was to compare the concentration of cardiac troponin I in the first morning urine in patients with severe aortic stenosis and the healthy population. **Patients and Methods**: Blood and first morning urine samples were collected from 34 healthy individuals (17 female) at University Hospital Merkur and 25 patients with severe aortic stenosis (14 female) before surgical treatment at University Hospital Dubrava. Cardiac troponin I and T values were determined using high-sensitivity assays using commercially available Abbott and Roche tests. **Results**: Patients with severe aortic stenosis had significantly lower troponin I concentrations in the first morning urine samples (0.3 ng/L (0.1–0.6)) as compared to the healthy population (15.2 ng/L (8.4–19.9)) (*p* < 0.001). There was no statistically significant difference in troponin T concentrations between healthy individuals and patients with severe aortic stenosis. In parallel, both I and T plasma troponin concentrations were significantly higher in patients with severe aortic stenosis. **Conclusions**: In patients with severe aortic stenosis, cardiac troponin I values in the first morning urine are significantly lower than in healthy subjects.

## 1. Introduction

Aortic stenosis (AS) is the most common acquired valvular heart disease in the Western world. As the population ages, degenerative aortic valve disease is a growing cause of cardiovascular disease in the elderly population [1]. Cardiac ultrasound is the most important tool for the assessment of aortic valve stenosis in patients with severe aortic stenosis due to its availability and reproducibility. It allows us to evaluate the valve itself (visualization of the valve, echogenicity of the valve, and estimation of the degree of degenerative changes) and analyze changes in hemodynamic parameters as a result of stenosis (measurement of blood flow velocity, aortic valve opening area, and pressure gradient) [2].

AS is a chronic progressive disease. In order to slow down the progression of aortic stenosis, optimal control of cardiovascular risk factors is required, the most important of which is arterial hypertension. Poor control of cardiovascular risk factors leads to faster progression of aortic stenosis [3]. Timely treatment of AS continues to be a medical challenge, although gradation according to the area of stenosis is used as the main parameter when deciding on an intervention. As aortic stenosis progresses to a greater degree, increasing pressures in the left ventricle are required for the heart to eject sufficient stroke volume and thus maintain an adequate cardiac output. Increasing pressures in the left ventricle cause hypertrophy of the left ventricle to enable more efficient heart function and to achieve adequate ejection volume. Increased hypertrophy of the left ventricle leads to increased metabolic demands and represents an energy burden on the myocardium itself. In such situations, the compensatory mechanism becomes insufficient after some time, and cardiomyocyte death and extracellular fibrosis gradually occur [4]. The size of extracellular myocardial fibrosis was shown to be an independent predictive factor of worse outcomes. Patients with severe aortic stenosis in whom the occurrence of extracellular myocardial fibrosis is verified using imaging methods (magnetic resonance) require urgent intervention such as surgery or transcatheter aortic valve implantation (TAVI) [5].

Until recently, cardiac troponins (cTns) T (cTnT) and I (cTnI) were exclusively used to rule out acute coronary syndrome. Today, they are also used in risk stratification for cardiovascular diseases [6]. Elevated blood troponin values in patients without clinical findings of acute coronary events indicate conditions that carry an increased cardiovascular risk. Values can be elevated in chronic kidney disease, chronic coronary syndrome, and other chronic conditions. Also, diagnostic tests that indicate potential dysfunction of cardiovascular functionality (e.g., intima/media thickness, ankle brachial index, and calcium score) correlate well with cardiac troponins in the blood [7].

Recent research that investigated the elimination of cTns indicates that it is possible to detect cTns in first morning urine using commercially available tests [8]. When measuring cTn concentrations, it appears that these concentrations in the first morning urine are several times higher than in the blood. Moreover, one research study showed that the concentrations of cTns in the first morning urine are significantly elevated in patients with increased cardiovascular risks (arterial hypertension) as compared to the healthy population [9]. Recently published papers indicate that urinary troponin values can be used as a prognostic factor for the development of cardiovascular incidents [10].

The aim of this research study was to investigate the difference in high-sensitivity (hs) cTn concentrations in the blood and in the first morning urine of patients with severe AS and healthy individuals.

## 2. Materials and Methods

Patients with AS. This study included patients who were hospitalized at University Hospital (UH) Dubrava for operative treatment of severe aortic stenosis from 1 June 2019 to 31 December 2019. Blood and first morning urine samples were collected from a total of 25 adult patients (14 females).

Healthy population samples. Samples from healthy participants were collected at UH Merkur from 1 June 2017 to 31 December 2017. The main aim of collecting samples from the healthy population was to create reference values for first morning urinary troponin concentrations. Among them, 34 (17 females) healthy volunteers had their blood and first morning urine samples collected for determination of cTnI and cTnT. All the collected samples were given to UH Merkur for storage and further analysis.

Methodology of troponin I determination. The Abbott Architect Troponin-I assay (Architect i1000SR Abbott, Paris, France) is a high-sensitivity, two-step immunoassay that is used to determine the presence of cTnI in human serum and plasma using chemiluminescence microparticle-immunoassay (CMIA) technology. In the first step, sample, assay diluent, and paramagnetic microparticles coated with anti-troponin I antibodies are combined. Troponin I present in the sample binds to the anti-troponin I-coated microparticles. After incubation and wash steps, the anti-troponin I acridinium-labeled conjugate is added in the second step. Following another incubation and wash, pre-trigger and trigger solutions are then added to the reaction mixture. The resulting chemiluminescent reaction is measured as relative light units (RLUs). A direct relationship exists between the amount of troponin I in the sample and the RLUs detected by the system’s optics. The concentration of troponin I is read relative to a standard curve established with calibrators of known troponin I concentrations. 

Methodology of troponin T determination. The Roche Elecsys Troponin T assay is a high-sensitivity sandwich electro-chemiluminescence immunoassay (ECLIA) that employs two monoclonal antibodies specifically directed against human cTnT (Roche Diagnostics, Mannheim, Germany). The antibodies recognize two epitopes (amino acid positions 125–131 and 136–147) located in the central part of the cTnT protein, which consists of 288 amino acids. Antigen in the sample, a biotinylated monoclonal cardiac troponin T-specific antibody, and a monoclonal cardiac troponin T-specific antibody labeled with a ruthenium complex react to form a sandwich complex. After the addition of streptavidin-coated microparticles, the complex becomes bound to the solid phase through the interaction of biotin and streptavidin. The reaction mixture is aspirated into the measuring cell where the microparticles are magnetically captured onto the surface of the electrode. Unbound substances are then removed. Application of a voltage to the electrode then induces a chemiluminescent emission, which is measured by a photomultiplier. The results are determined using a calibration curve, which is generated in an instrument-specific manner using 2-point calibration and a master curve. 

cTnT and cTnI concentrations were also determined in urine using the same protocol described above for their determination in serum or plasma [8,11].

Statistics. Data are presented as frequencies (n) and percentages (%) with mean ± SD (standard deviation) in cases in which the data are normally distributed or as medians with an IQR (interquartile range). The normality of the data distribution was tested using the Shapiro–Wilk test. The Fisher exact test was used to compare nominal data between the groups. At the same time, the independent sample *t*-test and Mann–Whitney U Test were used to compare normally and non-normally distributed variables, as appropriate. Spearman’s correlation analysis was used to analyze the correlations between variables. All data were analyzed using SPSS statistical software version 29.0. *p* values < 0.05 were considered statistically significant. 

## 3. Results

Anthropometric data as well as cTnT and cTnI concentrations in plasma and first urine samples for patients with AS and healthy individuals are presented in Table 1. Additional data describing the patients with severe AS are listed in Table 2.

The concentrations of cTnT and cTnI were significantly higher in the plasma of patients with severe AS as compared to healthy individuals [8.1 ng/L (5.4–17.5) vs. 0.9 ng/L (0.5–1.3) for TnI, *p* < 0.001; 15.2 ng/L (9.9–23.5) vs. 3.4 ng/L (3.0–5.5) for TnT, *p* < 0.001].

cTnI concentration in the first morning urine was significantly lower in patients with severe AS as compared to the healthy controls [0.3 ng/L (0.1–0.6) vs. 15.2 ng/L (8.4–19.9), *p* < 0.001]. cTnT concentrations in the first morning urine did not significantly differ between the observed groups (*p* = 0.454).

The concentrations of cTnI in the plasma samples of patients with severe AS were, on average, 27 times higher than in the first morning urine, while the concentrations of cTnT were, on average, only 1.8 times higher in the plasma. In parallel, a contrary observation was seen in healthy subjects, who presented with cTnI concentrations in the first morning urine that were, on average, 16.9 times higher than in the plasma. cTnT concentrations in healthy participants were, on average, 2.6 times higher in the first morning urine than in the blood, which is more similar to the results observed in severe AS patients. 

In healthy individuals, age had no influence on the concentration of cTnI in the first morning urine (Spearman’s r = −0.035, *p* = 0.845). The concentration of cTnT in the first morning urine showed a slight nonsignificant correlation with age (Spearman’s r = 0.198, *p* = 0.262). Age had no influence on the concentrations of cardiac cTnI (Spearman’s r = 0.081, *p* = 0.65) and cTnT (Spearman’s r = −0.083, *p* = 0.64) in the blood.

We performed a post hoc power analysis of our results. For the 25 patients in the aortic stenosis group and the 34 healthy individuals, the result of the power analysis was 0.79. Due to the significant difference in age between the groups, we performed a univariate binary logistic regression. The results are presented in Table 3.

Cardiac ultrasound in patients with severe aortic stenosis showed that, for the most part, the patients had preserved ejection fractions (EF) (median, 57%; IQR, 47.5–64%), indicating that left ventricular systolic function was maintained. Left ventricular end-diastolic volume (LVEDV) and left ventricular end-systolic volume (LVESV) were not increased; the median LVEDV was 118 mL (IQR, 103–158 mL), and the median LVESV was 53 mL (IQR, 42–72 mL). Peek jet velocities through the aortic valve were mostly above 4 m/s, (median, 4.54 m/s; IQR, 4.12–4.87 m/s). The maximal pressure gradient across the aortic valve was 82.88 mm Hg (median) with an IQR of 67.32–95.04 mm Hg, while the mean pressure gradient was 50.92 mm Hg (median) with an IQR of 40.45–64.21 mm Hg.

Renal function, according to estimated glomerular filtration rate (eGFR), in patients with severe aortic stenosis was normal for most of the patients (the median value was 81 mL/min/1.73 m^2^, with minimum and maximum observed values of 42 mL/min/1.73 m^2^ and 100 mL/min/1.73 m^2^, respectively). Renal function in these patients was determined using the CKD-EPI formula [12].

## 4. Discussion

The results of this study clearly indicate the potential of a low cTnI concentration in first morning urine samples to be a new specific biomarker in patients with severe AS, which is the main novelty of this research in the field of cardiovascular event prediction and diagnosis. Surprisingly, in all patients scheduled for aortic valve replacement, cTnI concentrations in the first morning urine were barely measurable, while in healthy individuals, cTnI concentrations in the first morning urine were several times higher than in plasma. Moreover, cTnT concentrations were not significantly different between groups. Our previous work indicated that the cTnI concentrations of a healthy population were several times higher in the first morning urine than in the plasma [8]. We also showed that in pathological conditions that have an impact on the heart, such as unregulated arterial hypertension and hypertensive urgency, the concentration of cTnI in the first morning urine was significantly higher as compared to the healthy population [9]. The results of this study are completely opposite to what we expected due to our previous studies. 

cTnT is a 34 kDa molecule. As soon as it reaches the blood, it degrades into smaller fragments that can still be detected with commercially available tests. The two most common fragments are 24 kDa and 17 kDa [13]. cTnI is a molecule with a size of about 24 kDa, and, in the blood, it is found predominantly in two fragments of about 21 kDa and 14 kDa that can also be detected using commercially available tests [14]. While cTnT has a slight negative charge (pI around 5.1), cTnI is a positively charged molecule (pI of 9.8) and is most often found in the circulation in a form that is associated with cTnC [15]. This difference in polarity between the cTnT and cTnI molecules is most pronounced in hemodialysis-dependent patients. Due to its biochemical properties, cTnI is mainly bound to the dialysis membrane [16], and its concentration in the dialysate is minimal, but cTnT freely passes into the dialysate, and its concentration in the dialysate is significantly higher [17]. 

Using tests of the latest generation, cTns can be detected in the blood even at nano concentrations. In healthy individuals, a stable concentration of cTns in the blood is maintained by the constant replacement of proteins in the heart and their clearance by the kidneys, which enables their detection in the urine [9]. In pathological conditions such as acute coronary syndrome, there is a sudden release of a large quantity of cardiac proteins. cTns are then mainly found in the blood in the form of a macromolecule (multiple troponin units connected within one protein complex), which is predominantly removed by the reticuloendothelial system [18,19]. Smaller molecules pass through the glomerular membrane more easily and can therefore be detected in the urine. Their renal clearance depends on their size, polarity and interaction with other protein molecules in the blood. Considering all previously mentioned factors, the concentrations of different protein molecules in the first morning urine can differ significantly [18]. This is the first study that showed measured concentrations of cardiac troponins T and I in the blood and first morning urine in a healthy population.

Compensatory hypertrophy of the left ventricle occurs in patients with severe AS. Having to overcome higher pressure, the cardiomyocytes thicken, causing hypertrophy of cellular contractile elements. This increased volume of cardiomyocytes causes increased energy consumption and mitochondrial dysfunction, which leads to further cellular energy insufficiency [20]. The need for cardiomyocytes to generate stronger contractile forces increases cellular calcium levels. Due to energy deprivation in cardiomyocytes, the apoptotic effects of high cellular calcium levels on cardiomyocytes occur [21]. All this leads to an increased turnover of cellular proteins and the cardiomyocytes themselves due to the load. Excessive use of cellular energy leads to a weakening of heart function, and signs of heart failure appear. Initially, these signs include increased left ventricular filling pressure, and later, significant cardiac dysfunction occurs. 

Under normal conditions, cardiac troponins are gradually replaced by new molecules. Older molecules are released into the blood, which is one of the reasons that enables the measuring of the concentrations of cardiac troponins in healthy individuals. Molecules found in the blood of healthy individuals are generally of lower molecular mass than complete troponin molecules (troponin degradation products) [22]. Such breakdown products of troponin are eliminated from the blood predominantly by the kidneys [18]. 

In situations of increased myocardial stress that cause cardiomyocyte damage and death, such as myocardial infarction or severe aortic stenosis, there is an increased release of cardiac troponins into the blood as part of cardiomyocyte injury/death [13]. The troponin that is released from cardiomyocytes into the blood in these situations has different biochemical characteristics [23,24]. Due to the complexity of the breakdown products of the cTnI molecule in the blood, there is a possibility that these products will bind to larger troponin complexes (composed of troponin T, I, and C), and the clearance of these complexes by the kidneys may be reduced [15]. In these situations, the dominant route of troponin elimination from the blood is the reticuloendothelial system [18,25]. We think this is most probably the main reason why cTnI concentration in the first morning urine samples of patients with severe aortic stenosis is reduced.

Much is still unknown about troponin elimination pathways and its biochemical characteristics, and this is an area of intense research. We believe that the above pathophysiological mechanism could correspond to the actual conditions found in the human body.

Chen et al. analyzed the predictive values of urinary cTnI. They showed that higher concentrations of cTnI in urine are associated with a higher possibility of an acute cardiac event. They also indicated that cTnI concentrations of 4.1 pg/mL in urine samples have predictive values for future cardiovascular events [10]. A significant proportion of the patients in that study were heart failure patients. AS is one of the most common valvular diseases that can cause heart failure. Concentrations of cTnI in the first morning urine (which is known to be more concentrated) in patients with severe AS in our study were significantly lower than 4.1 pg/mL, as suggested in Chen et al. Therefore, when considering our results and the Chen et al. paradigm, patients with severe AS who have an increased risk of acute cardiac events such as a heart failure would be misevaluated if urine cTnI was used as a prognostic biomarker of future cardiac events, as we are proposing in this research study. 

Numerous biomarkers have been investigated as prognostic markers in patients with severe aortic stenosis. A significant portion of them are cardiac-specific, such as NT-proBNP, hsTnI, and hsTnT, while others are specific for some specific conditions such as inflammation and accelerated atherosclerosis. Baran et al. observed cardiovascular outcomes in patients with severe aortic stenosis who underwent an intervention to correct the aortic valve (either surgery or transcatheter valve replacement) and found that the values of endothelin 1 and galectin-3 showed a significant correlation with cardiovascular mortality and progression of heart failure [26]. Also, numerous studies of the progression of aortic stenosis due to epigenetic changes in the aortic valve itself have shown that it is possible to use new epigenetic markers (microRNA, lncRNA, etc.) in the diagnosis of aortic stenosis [27]. So far, the optimal biomarker that would allow us to intervene in a timely manner to correct the aortic valve has not yet been discovered.

Low concentrations of troponin I in the first morning urine could be a good indicator of patient follow-up after intervention (either surgery or transcatheter valve replacement) to correct the aortic valve. Additional research is needed to investigate the dynamics of urinary troponin I elimination in patients who have had an intervention to correct the aortic valve.

Although our research pointed out some novelty in the field of cardiovascular medicine, it has a few limitations that need to be highlighted. The main limitation of this study is that the experimental and control groups significantly differed in age. Patients with severe aortic valve stenosis are mostly of an older age, considering that degenerative valve disease is the main cause of AS. On the other hand, it is extremely difficult to find healthy individuals who do not have any comorbidities and are of an older age. However, it should be taken into account that our group of healthy individuals included only individuals who did not have any comorbidities and were not under any medical treatment at the moment of their inclusion in this research study. cTnI and cTnT have been measured with commercially available high-sensitivity assays in both plasma and urine samples. The main limitation of these tests is that they are certified for measuring cTn concentrations either in serum or plasma samples for diagnostic purposes. However, since there are no currently available assays that are certified to measure urine cTn concentrations, we believe that assays with characteristics such as limit of blank (LoB) are good enough to measure cTn concentrations in urine samples with sufficient precision and accuracy. Earlier works indicated that this method is applicable [8,10,11]. 

One of the most significant limitations of the study is the small number of participants in the groups. To make more precise clinical decisions, it is necessary to examine the influence of troponin I in the first morning urine in a significantly larger number of patients with severe aortic stenosis. The difference in the concentration of troponin I in the first morning urine between patients with severe aortic stenosis and healthy individuals was statistically significant despite the small number of participants in the groups.

Although the groups differed in age, we believe that an extremely low concentration of cardiac-specific troponin I in the first morning urine samples is a unique peculiarity of the group of patients with aortic stenosis. 

It is possible that the altered biochemical characteristics of cTnI indicate the beginning of negative remodeling of the myocardium. This could be an early signal of irreversible changes in the heart in patients with severe aortic stenosis. This altered cTnI could be the main reason for its reduced clearance by the kidneys and its reduced concentration in the first morning urine samples. 

One of the main pathophysiological findings in patients with severe aortic stenosis is the appearance of myocardial fibrosis, which can only be detected and quantified using heart MR [5]. Given that the aforementioned test is not readily available to the entire population, further research is needed to find simpler and cheaper diagnostic tools that will be able to show a good correlation with myocardial fibrosis. One of these potential diagnostic markers could be the measurement of cTnI in the first morning urine.

Further research is needed to find out if different stages of AS (moderate and mild aortic valve stenosis) could have different impacts on the dynamics of cardiac troponin I concentration in first morning urine samples. Furthermore, the impact of renal function on cTnI concentrations in the first morning urine should also be investigated.

## 5. Conclusions

A significantly lower concentration of cardiac troponin I in the first morning urine was found in patients with significant stenosis of the aortic valve compared to the healthy individuals. Therefore, low concentrations of troponin I in the first morning urine in patients with aortic stenosis indicate that these are patients who need either an intervention to correct the aortic valve or further cardiological examination.

## Figures and Tables

**Table 1:** Basic data from aortic stenosis patients and healthy controls as well as plasma and first morning urine cardiac troponin concentrations.

Variable	AS	CONTROL	*p*-Value
	*n* = 25	*n* = 34	
Gender			0.797 *
male	12 (44.4)	17 (50.0)	
female	15 (55.6)	17 (50.0)	
Age (y)	70.5 (64–75)	34.0 (28.0–44.0)	<0.001 **
Height (cm)	165.0 ±10.4	176.2 ± 11.65	<0.001 ^x^
Weight (cm)	84.28 ± 14.38	78.07 ± 18.40	0.167 ^x^
BMI kg/m^2^	31.04 ± 4.99	24.81 ± 3.51	<0.001 ^x^
BSA (m^2^)	1.91 ± 0.18	1.95 ± 0.29	0.499 ^x^
hsTnI plasma (ng/L)	8.1 (5.4–17.5)	0.9 (0.48–1.33)	<0.001 **
hsTnI urine (ng/L)	0.3 (0.1–0.6)	15.2 (8.4–19.9)	<0.001 **
hsTnT plasma (ng/L)	15.18 (9.96–23.49)	3.4 (3.0–5.5)	<0.001 **
hsTnT urine (ng/L)	8.38 ± 2.51	8.94 ± 3.13	0.454 ^x^

AS: aortic stenosis patient group, BMI: body mass index, BSA: body surface area, hsTnI: high-sensitivity troponin I, hsTnT: high-sensitivity troponin T, * = Fisher exact test, ** = Mann–Whitney U Test, ^x^ = Independent sample *t*-test.

**Table 2:** Additional data from severe aortic stenosis patients.

	YES	NO
Smoking history	6	19
Arterial hypertension	20	5
Diabetes	6	19
Dyslipidemia	15	10
CVI	5	20
ACEi	18	7
CCB	13	12
BB	14	11
Statins	10	15
Diuretics	13	12

CVI: cerebrovascular insult, ACEi: angiotensin-converting enzyme inhibitors, CCB: calcium channel blockers, BB: β-blockers.

**Table 3:** Univariate binary logistic regression of troponin T and I in plasma and first morning urine with incidence of aortic stenosis.

Variable	OR	OR (95% CI)		*p*
plasma hsTnT	1.661	1.266	2.178	<0.001
urine hsTnT	0.933	0.776	1.121	0.459
plasma hsTnI	2.734	1.642	4.552	<0.001
urine hsTnI	2.1794 × 10^−26^	0.000	-	0.964

hsTnI: high sensitivity troponin I, hsTnT: high sensitivity troponin T.

## Data Availability

The data presented in this study are available on request from the corresponding author due to the fact that the data presented here are the part of an ongoing study.
